# Current Advances of Nanomedicines Delivering Arsenic Trioxide for Enhanced Tumor Therapy

**DOI:** 10.3390/pharmaceutics14040743

**Published:** 2022-03-30

**Authors:** Mengzhen Yu, Yanwen Zhang, Meirong Fang, Shah Jehan, Wenhu Zhou

**Affiliations:** Xiangya School of Pharmaceutical Sciences, Central South University, Changsha 410013, China; 8305180309@csu.edu.cn (M.Y.); zhangyanwen@csu.edu.cn (Y.Z.); 8305180213@csu.edu.cn (M.F.)

**Keywords:** Arsenic trioxide, nanomedicines, bioavailability, cytotoxicity, synergistic effect, combinatorial treatment

## Abstract

Arsenic trioxide (ATO) is one of the first-line chemotherapeutic drugs for acute promyelocytic leukemia. Its anti-cancer activities against various human neoplastic diseases have been extensively studied. However, the clinical use of ATO for solid tumors is limited, and these limitations are because of severe systemic toxicity, low bioavailability, and quick renal elimination before it reaches the target site. Although without much success, several efforts have been made to boost ATO bioavailability toward solid tumors without raising its dose. It has been found that nanomedicines have various advantages for drug delivery, including increased bioavailability, effectiveness, dose-response, targeting capabilities, and safety as compared to traditional drugs. Therefore, nanotechnology to deliver ATO to solid tumors is the main topic of this review, which outlines the previous and present medical applications of ATO. We also summarised ATO anti-cancer mechanisms, limitations, and outcomes of combinatorial treatment with chemo agents. As a result, we strongly recommend conducting pre-clinical and clinical studies of ATO, especially nano-system-based ones that might lead to a novel combination therapy for cancer treatment with high efficacy, bioavailability, and low toxicity for cancer patients.

## 1. Introduction

Malignant tumors have always posed a threat to human health, and their morbidity and fatality rates have been rising year after year [1]. In 2020, according to the World Health Organization (WHO), an estimated 19.3 million new cancer cases were diagnosed (excluding non-melanoma skin cancer), and approximately 10million people died due to cancer (excluding non-melanoma skin cancer) [2]. Despite the massive investments in cancer treatment and prevention, cancer remains one of the major causes of death globally [3]. The main clinical treatment of malignant tumors is surgery, radiotherapy, chemotherapy, targeted therapy, endocrinotherapy, and immunotherapy, but it is difficult to achieve a satisfactory prognosis [1,4]. However, these therapies’ limitations include the off-target effect and suboptimal pharmacokinetics [5]. Some drawbacks of anti-cancer drugs include high cost, low bioavailability, limited efficacy, long-term treatment, and high toxicity [3,6]. Exploration and evaluation of non-cancer drugs for anti-cancer activity provides an opportunity to develop a new therapeutic strategy for cancer treatment [3]. This approach, alternatively called new uses for old drugs, drug repurposing and therapeutic switching, has gained considerable attention over the past decade [3].

For thousands of years, metals and metal compounds have been employed in medicine [7]. Arsenic compounds present a rich history in medicinal applications dating back to more than 2400 years [8]. Arsenic trioxide (ATO) was used in traditional Chinese medicine to treat syphilis, psoriasis and rheumatism [8]. In the modern era, active ingredients of arsenic compounds are orpiment (As_2_S_3_), realgar (As_4_S_4_), and ATO. These ingredients have been evaluated against various cancers, including acute promyelocytic leukemia (APL), myelodysplastic syndrome, multiple myeloma, and some solid tumors, including hepatocellular carcinoma, bladder, glioma, breast and renal cancers [9]. Specifically, the clinical dosage form of ATO is 5–10 mg via intravenous injection. Although ATO presents promising antitumor effects, the clinical application in tumors has been restricted by several factors, including severe side effects, low drug solubility and rapid renal clearance [10]. The optimization of ATO-based antitumor agents is divided into direct and indirect ways. The direct way is to improve the pharmacokinetic parameters of ATO by pharmaceutics methods, such as prolonging the circulation time and reducing clearance. The indirect way to enhance the antitumor effect of ATO is through combinations with other drugs. We summarized the relevant research over the last five years. We found that both pharmaceutics and drug combinations methods effectively improved the therapeutic potential of ATO.

Recently, the introduction of nanomedicines as innovative drug delivery systems (DDSs) enhanced the chemotherapeutics potency. These enhancements include increased bioavailability, reduced adverse effects and selectively killed tumor cells [11]. The enhanced permeability and retention (EPR) effect is the main advantage of nanomedicines [12]. This advantage provides the basis for the sufficiently killed tumor cells. Much attention has been drawn to nanoparticle systems to deliver inorganic arsenite or arsenous acid, which are highly toxic to normal tissue and almost insoluble [13]. But there is still no review about the recent progress of nanomedicines to deliver ATO for tumor therapy. Therefore, we summarized the common strategies to deliver ATO by nanoparticles. We also introduced the antitumor mechanisms of ATO and their possible combinations with other chemo agents for tumor therapy. Overall, we described ATO and provided information on their modernization.

## 2. Pharmacological Properties of ATO

### 2.1. Physicochemical Properties

**Figure 1:**
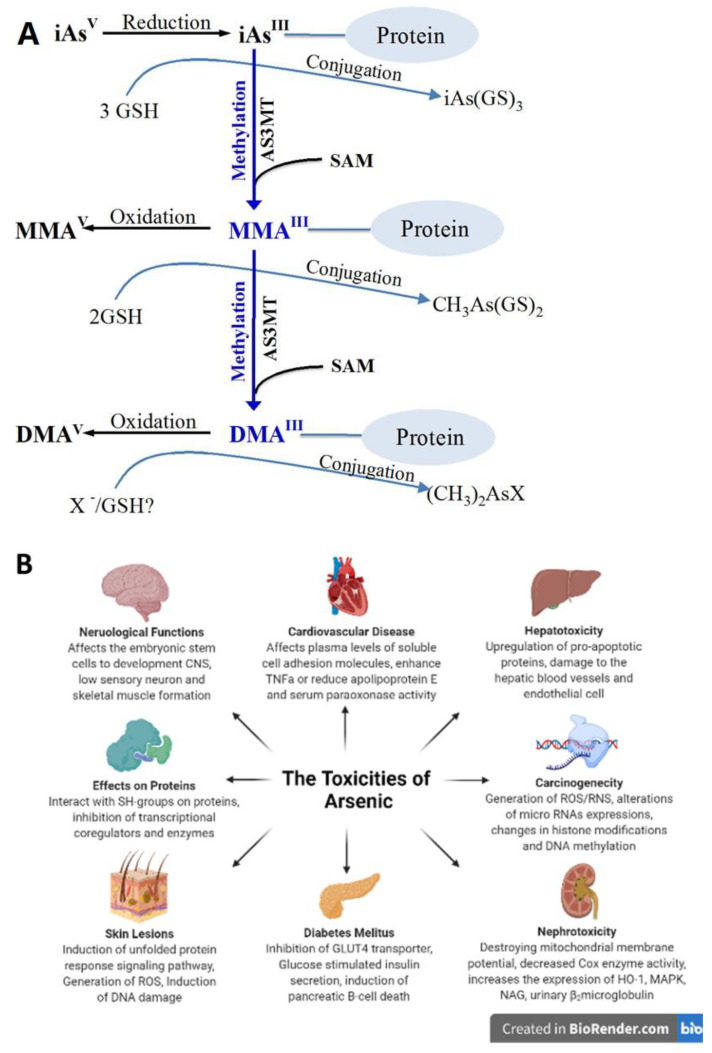
(**A**) Proposed arsenic metabolism pathway in human. (**B**) The schematic diagram represents the toxicities of arsenic compounds. Reproduced from ref. [16], Impact Journals, 2017.

The physicochemical properties of ATO are essential for their biological applications, such as bioavailability, biosafety, and therapeutic activities. Arsenic is a metalloid element having both metallic and non-metallic chemical properties that is found in the Earth crust [14]. Arsenic can produce inorganic derivatives when it reacts with sulfur and oxygen and organic compounds when it reacts with carbon and hydrogen [14]. Based on their formation, arsenic compounds can generally be divided into three categories with different toxicity: (1) inorganic arsenic compounds (iAs); (2) arsenic gas, which is recognized as highly toxic; and (3) organic arsenic compounds (oAs) that are relatively non-toxic (Figure 1) [15]. Inorganic arsenic compounds are recognized as a carcinogen by the International Agency for Research on Cancer (IARC) for lungs, bladder and skin [15].

Another essential parameter affecting its toxicity is the oxidation state, which can exist at −3, 0, +3 and +5 in nature [14]. Pentavalent forms of arsenic with the 5th oxidation state, such as AsV arsenic (As(V)) and trivalent arsenic (As(III), are the primary inorganic forms of arsenic compounds [17]. In general, trivalent meta-arsenite is more toxic than the pentavalent arsenate. This high toxicity is because of its capacity to interrupt proteins via high binding affinity with thiol groups [17]. ATO is highly toxic among all these inorganic forms, an industrial byproduct when roasting arsenic-containing ores. But elemental arsenic is almost not toxic [8,18].

### 2.2. Anti-Cancer Mechanisms

**Figure 2:**
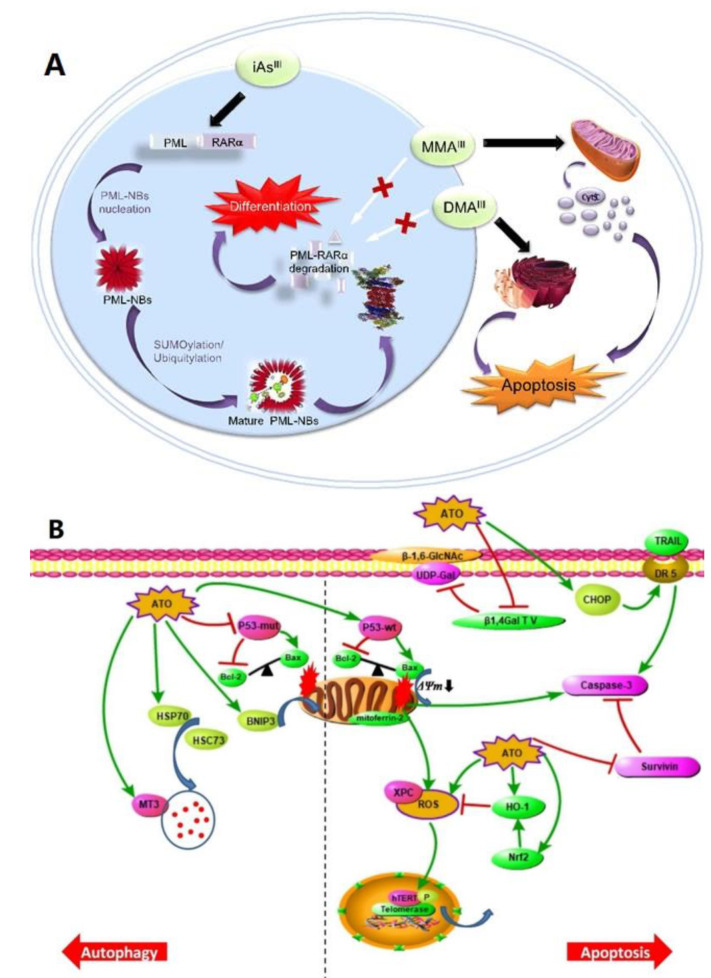
(**A**) Molecular mechanisms of iAs^III^ and its trivalent methylated metabolites (i.e., MMA^III^ and DMA^III^) to induce cell apoptosis in APL cells. Reproduced from ref. [16]. Copyright 2017 Oncotarget. (**B**) The mechanisms of glioma cell death induced by ATO. Reproduced from ref. [18], Springer Nature, 2020.

ATO has a long and illustrious history in medicine, stretching back over 2400 years. It was utilized in traditional Chinese medicine to treat rheumatism, psoriasis and syphilis [8]. In 2000, the US Food and Drug Administration (FDA) approved ATO Injection (Trisenox^®^, Cell Therapeutics, Inc., Seattle, WA, USA) as a crucial chemotherapeutic against acute promyelocytic leukemia [19]. Recently, ATO has become a highly potential antitumor drug, and the mechanisms have been studied extensively (Figure 2). ATO clinical effectiveness is attributed to several mechanisms. These mechanisms include the degradation of the APL-specific PML/RARα fusion transcript and apoptosis induction in promyelocytic cells by modulating Bcl-2, Bax proteins and elevated ROS levels [20,21]. In addition, ATO has been reported to interfere in other cellular events such as tubulin polymerization, DNA repair, and cell cycle progression [20]. ATO can also exert anti-cancer effects via inhibiting tumor stem cells and angiogenesis and enhance the effects of radiotherapy and chemotherapy [18]. Besides, more recent data suggest that ATO eliminates leukemic stem cells in APL [21]. ATO-induced deactivation of PML-RARα causes malignant promyelocytes to differentiate [22,23]. ATO anti-cancer properties are not limited to only APL. Other malignancies such as lung, prostate, breast, liver and stomach cancers have been proven to be inhibited by ATO in numerous studies over the last decade [24]. Apoptosis appears to be a prominent phenomenon, resulting in significant cell death and tumor growth inhibition [25]. Therefore, apoptosis is among the most common mechanisms for counteracting or sensitizing cancer cells to chemotherapeutic agents or radiation therapy [26].

There are two common signaling pathways for apoptosis regulation. When death ligands and death receptors bind to each other on the plasma membrane, the extrinsic pathway is initiated [27]. Oxidative stress damages mitochondrial integrity in the intrinsic pathway, causing mitochondrial dysfunction and cell death [28]. Several death ligands must be present for the extrinsic pathway to be activated, including the tumor necrosis factor-related apoptosis-inducing ligand (TRAIL), tumor necrosis factor (TNF), TWEAK and Fas ligand (FasL), and the accompanying death receptors, including DR3, TNF-R1, Fas and TRAIL-R1/-R2 [28]. A contact between FasL and a transmembrane receptor known as Fas can activate caspases 8 and 3, leading to apoptosis [28]. Wang GB et al. investigated whether ATO inhibits glioma cell proliferation and induced apoptosis via down-regulation of expressions of Fas and FasL [29]. For cancer cells, the proapoptotic effect of the TRAIL protein is facilitated by its interaction with death receptors, including DR4 (TRAIL-R1) and DR5 (TRAIL-R2) [28]. The upregulation of TRAIL expression directly inhibits tumor initiation and metastasis [30]. The present study of Wang LY et al. demonstrated that ATO combined with sorafenib induced HCC-cell death via the TRAIL signaling pathway [30].

The intrinsic signals of apoptosis mainly include interference of redox homeostasis. Redox homeostasis mainly depends on the ratio of antioxidants and oxidants such as glutathione (GSH) and reactive oxygen species (ROS), respectively. ROS plays a critical role in modulating oxidative stress [31]. Many studies have indicated that chemo agents interfering with ROS metabolism can disturb the intracellular oxidant and antioxidant balance that selectively eradicate the cancer cells by elevating ROS levels [26,28]. A previous report showed that ATO caused apoptosis in lung cancer cells by depleting GSH, reducing Trx, and generating ROS [28]. Once ROS accumulation occurs, the mitochondria are highly susceptible to oxidative damage. Thereby, the Bcl-2 protein exerts its anti-apoptotic function by reducing intracellular ROS. Interestingly, ATO downregulated Bcl-2 and promoted apoptosis in cancer cells [32].

The Bcl-2 protein is considered an important anti-apoptotic member of the Bcl-2 family proteins. Depending on the cellular context, its expression manifests either cytoprotective or cytodestructive phenotypes [25]. The intrinsic or mitochondrial apoptosis pathway is associated with the downregulation of BCL2 gene and upregulation of BID, BAX and/or BAK genes that depolarize the mitochondrial membrane. The depolarization of the mitochondrial membrane leads to the release of cytochrome C from mitochondria into the cytoplasm. Released cytochrome C binds to Apaf1 (apoptotic protease-activating factor 1) proteins that form the apoptosome complex to further activate caspases and eventually induce apoptosis [28,33]. Sun Y et al. showed that ATO induced apoptosis through the down-regulation of Bcl-2 and the up-regulation of Bax genes [32]. It has been reported that ATO caused apoptosis in lung cancer H841 cells by the downregulation of XIAP and the release of SMAC during treatment [34].

Autophagy has been recognized as a conserved intracellular degradation system that plays a vital role in many physiological and pathophysiological processes [35]. According to new research, autophagy appears to play a dual role in cancer, either saving cells from death or contributing to cell death [36]. A previous report showed that via the ROS-TFEB pathway, ATO causes osteosarcoma cell death by the induction of excessive autophagy [37]. Many reports showed that arsenic compounds induced apoptosis and activated autophagy [38]. Wang GY et al. found that As_2_S_2_ induced autophagy by activating ROS/JNK and inhibiting Akt/mTOR signaling pathways [35]. Interestingly, using autophagy inhibitors, 3-MA enhanced As_2_S_2_-induced cell death, indicating that As_2_S_2_-induced autophagy is a pro-survival process in cells [35]. Another study showed that autophagy inhibitors combined with ATO significantly inhibit cancer cell proliferation [39].

The anti-angiogenesis effect is the most critical therapeutic potential of ATO, among the recognized mechanisms of its anti-cancer effects [23,40]. Angiogenesis in tumors begins with the breakdown of the extracellular matrix (ECM) induced by matrix metalloproteinase (MMPs). Furthermore, platelet-derived growth factor (PDGF)/PDGFR-induced endothelial cell migration. As a result, fibroblast growth factor-2 (FGF-2)/FGFR-2 and vascular endothelial growth factor (VEGF)/VEGFR-2 promote cell proliferation, while delta-like canonical Notch ligand 4 (Dll4)/Notch-1 promotes vascular tube formation [41,42]. Zhang L et al. showed that ATO inhibited gastric cancer cell proliferation in a dose-dependent manner and significantly decreased tumor growth and angiogenesis by upregulating FOXO3a expression in mice [43]. ATO can inhibit FoxO3a phosphorylation, and this inhibition will promote its nuclear translocation. FoxO3a knockdown reduced the ATO anti-angiogenesis activity [24]. Lou DH et al. investigated the role of ATO in ovarian cancer angiogenesis and found that ATO inhibits VEGFA and VEGFR2 expression, thereby inhibiting the VEGFA-VEGFR2-PI3K/ERK signaling pathway [44]. Song P et al. showed the underlying mechanisms of realgar transforming solution (RTS) for anti-angiogenesis; results demonstrated that RTS had a strong activity to inhibit human umbilical vein endothelial cells (HUVECs) proliferation, angiogenesis, invasion and migration [45]. Moreover, RTS also decreased the phosphorylation of VEGFR2 by VEGF/bFGF and the downstream protein kinases ERK, Src and FAK [45]. Apart from the VEGF pathway, the Notch pathway has been identified as an essential regulator of angiogenesis. Some researchers suggested that the antiangiogenic effect of ATO was mediated through the blockade of the Notch signaling pathway, probably due to targeting the Notch1 protein [43].

The cell cycles G1, S, G2, and M phases play a crucial role in cell differentiation, proliferation, apoptosis and DNA damage repair [28]. An uncontrolled cell cycle is one of the leading causes of cancer. Therefore, the drugs that induce cancer cell cycle arrest have become the focus in cancer treatment [28]. An increasing array of research has elucidated that arsenic compounds serve as anti-cancer agents by inducing tumor cell cycle arrest at the G2/M phase [28,35,46,47,48,49]. Laka Kagiso et al. investigated that ATO inhibited the proliferation and affected the morphology of the MCF-7 breast cancer cells. They also discovered that ATO induced G2/M cell cycle arrest and caspase-dependent death in MCF7 cells without causing mitochondrial membrane disruption [47]. Hassani S et al. suggested that the demethylation of cyclin genes by ATO such as CCND1 and CCNE1 accelerates G1 and S transition into the G2/M cell cycle arrest [49].

Many reports have shown that cancer stem cells (CSCs) play a critical role in sustaining the malignant phenotype. The CSCs are responsible for the poor prognosis and resistance to radiotherapy and chemotherapy. Therefore, many chemotherapeutics kill the malignant tumor bulk population, but CSCs can survive and cause recurrence [28,50]. Ning K et al. reported that ATO decreased tumor growth, the conversion from colorectal CSCs to CRC cells, and augmented the density of CSCs [51]. The function of CSCs in lung cancer is maintained through signaling pathways such as WNT, Notch, and Hedgehog [28]. ATO has been reported to inhibit Gli1, a key transcription factor of the Hedgehog pathway [52,53]. Linder, B et al. demonstrated that the ATO combined with Gossypol synergistically inhibited cell proliferation via modulating the Hedgehog/Notch pathway [54]. Besides, mini-chromosome maintenance protein (MCM) 7 was recognized as a potential target that was down-regulated dramatically by ATO [55]. Moreover, MCM7 silencing recapitulates the effects of ATO on metastasis and CSCs, Indicating that ATO may affect CSCs by targeting MCM7 protein [55].

## 3. Limitations of ATO for Clinical Applications

Although arsenic compounds present a promising antitumor effect, their clinical application in solid tumors has been restricted by several factors, such as low drug solubility and rapid renal clearance [13,56]. High-dose or chronic exposure to arsenic compounds is associated with severe adverse effects, including impaired neurological activities, cardiovascular disease, diabetes, and cancer incidence [15]. Literature has shown that ATO could induce many types of side effects, including skin lesions (23%), gastrointestinal symptoms (24%) such as diarrhea, nausea, vomiting, dyspnea, eczema, headache and fever [57]. Arsenic compounds have been implicated in various neurological diseases, such as Alzheimer’s and Parkinson’s disease, via their ability to form plaques, enter the substantia nigari, and affect dopaminergic brain function [58]. And the International Agency for Research on Cancer (IARC) and the Food and Drug Administration (FDA) have demonstrated that arsenic compounds increase the risk of tumors of the bladder, lungs, kidneys and liver [59]. The mechanisms for arsenic-induced carcinogenesis are not fully understood [60]. Several modes of action of arsenic compounds have been reported, including increased oxidative stress associated with low levels of SOD1, DNA repair inhibition, and chromosomal alterations [61].

Besides the cytotoxicity, the renal clearance of most arsenic compounds is rapid, which leads to a short half-life in plasma and low drug concentration around tumor sites [62]. But a high dose of ATO will induce severe adverse reactions, such as liver dysfunction, skin reactions, and cardiac toxicity. A study found that a dose of more than 0.20 mg ATO/kg/d may cause severe side effects, including flaccid paralysis and renal failure [63]. ATO presents promising therapeutic effects based on the properties mentioned above, but it is still challenging to use in clinical applications. Therefore, the strategies to enhance the efficacy with the safety of arsenic compounds becomes an urgent question.

## 4. Strategies to Deliver ATO by Nanoparticles

**Figure 3:**
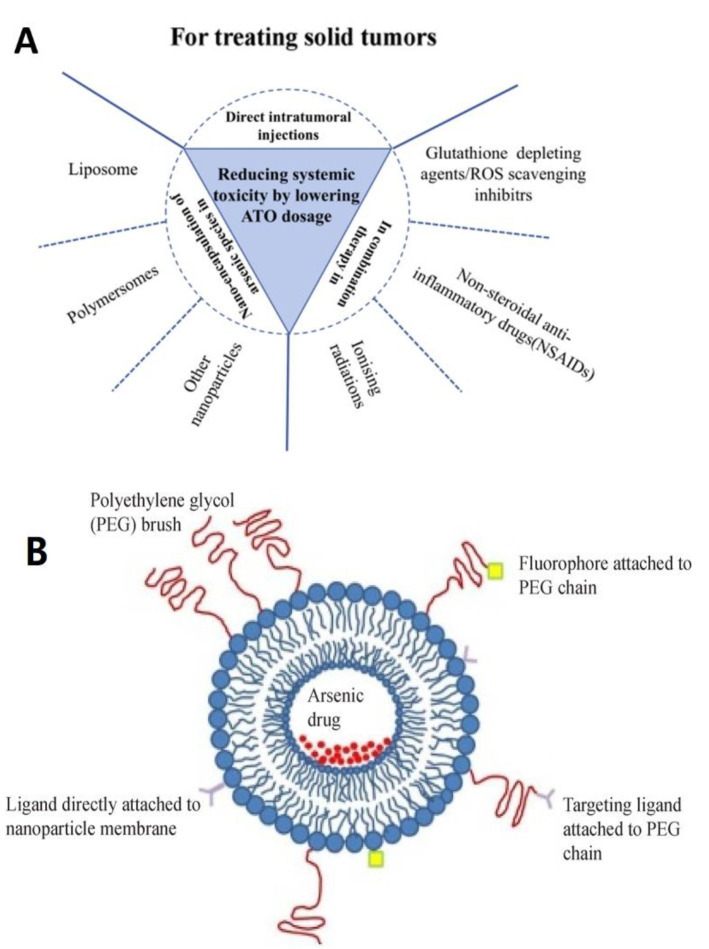
(**A**) Key therapeutic strategies employed to boost the efficacy of ATO as a drug in treating solid tumors without escalating its dosage. (**B**) Applications of nanotechnology for ATO drug delivery. Reproduced from ref. [8], Nanjing Medical University, 2017.

The rapid advancement of nanomedicine could lead to improved treatment efficacy and reduced chemotherapeutic toxicity [64]. The unique enhanced permeability and retention (EPR) is the main advantage of nanoparticles [65]. Many nanoparticle formulations improved the therapeutic potential of arsenic compounds, including drug loading efficiency, drug selectivity to the target site, and drug accumulation rate in tumor (Table 1 and Figure 3) [13].

### 4.1. Liposome

**Figure 4:**
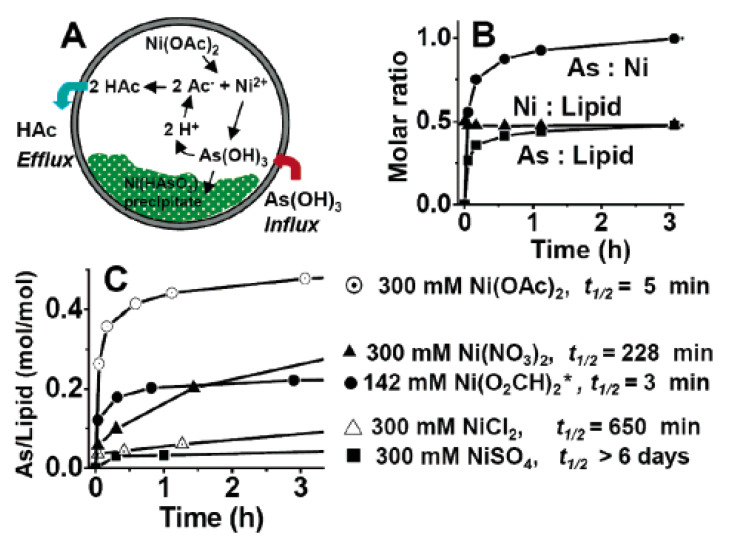
(**A**) Schematic representation of arsenic loading mechanism. (**B**) Molar ratios of As/lipid, Ni/lipid, and As/Ni as a function of incubation time during arsenic loading into liposomes at 50 °C using 300 mM Ni(OAc)_2_ (pH 6.8) as an intraliposomal medium. (**C**) Kinetics of arsenic loading into liposomes using various Ni(II) solutions as the intraliposomal medium at pH 6.8; *, the low solubility of Ni(II) formate prevented its examination at 300 mM. Reproduced with permission from ref. [77], American Chemical Society, 2016.

Drug delivery to tumors via liposomal carriers has been extensively studied. This delivery maintained drug stability, prolonged blood circulation, improved antitumor efficiency, and reduced the side effects of chemotherapeutics [63]. Encapsulating ATO in liposomes also enhanced the drug therapeutic index by reducing its side effects and increasing drug concentration in tumors through an EPR effect (Figure 4) [73]. A previous report showed that ATO incorporated in liposomes exhibited augmented toxicity to tumor cells while being less toxic to healthy cells [74]. Zhao et al. prepared liposomes containing ATO and administered them intravenously to rats to evaluate their anti-cancer effect against C6 glioma cells. The result showed that liposomal preparation led to a five-fold increase in ATO in rat brains as compared to single treatment, triggering apoptosis and reducing tumor angiogenesis by interfering with the expression of vascular endothelial growth factor (VEGF) with low toxicity [75]. Chen et al. demonstrated using folic acid as a targeting ligand to introduce ATO into tumor cells which overexpressed folic acid receptors (FR). Such “targeted” liposomes were tested on FR positive KB (human nasopharyngeal) cells, and the cellular absorption of ATO via FR-mediated endocytosis was reported to be substantially higher than FR negative MCF-7 (breast cancer) cells, attaining three to six times higher uptake than untargeted ATO liposomes [66].

Hengwu Xu et al. found that angiopep-2-modified calcium arsenite-loaded liposomes (A2-PEG-LP@CaAs) have a high drug-loading capacity and entrapment efficiency. ATO was responsively released in the acid tumor microenvironment, thereby exerting an anti-glioma effect. The lipoprotein receptor-related (LRP) receptor, which is overexpressed in the blood-brain barrier (BBB) and glioma, is responsible for this specific anti-glioma effect. As a result, A2-PEG-LP@CaAs might significantly enhance ATO anti-glioma activity, making it a promising glioma therapeutic strategy [67].

In addition, the metal-arsenic complex has been raised due to its high loading efficiency of ATO. A previous report showed that actively incorporating ATO into liposomes was a successful strategy to improve the loading efficiency. This combination also enhanced the metal-arsenic complex’s stability and prolonged the in vivo circulation [76]. Shaoning Wang et al. encapsulated ATO in liposomes via copper acetate (Cu (OAc)_2_) gradients, and a high entrapment efficiency of over 80% was obtained. The tissue distribution and pharmacokinetics tests of ATO liposomes revealed a considerably lower plasma clearance rate and enhanced T1/2 and AUC 0–12 h. It was found that ATO-loaded liposomes boosted the anti-cancer effect on S180 tumor-bearing mice by 61.2%. The toxicity of ATO was considerably lowered when it was encapsulated. In summary, the remote loading approach using Cu(OAc)_2_ gradients can successfully encapsulate ATO into liposomes. Liposomal formulations combining ATO and Cu(II) could be useful in treating a wide range of malignancies [63].

### 4.2. Protein

**Figure 5:**
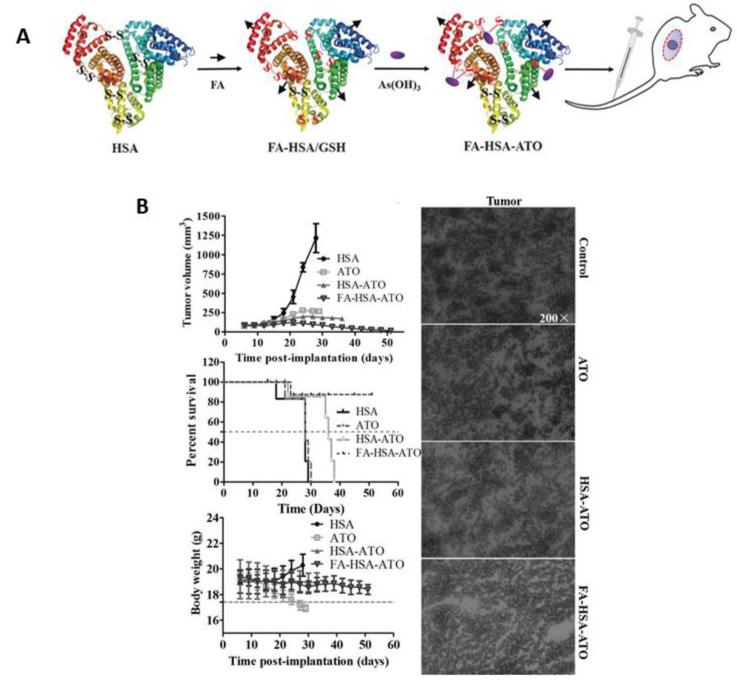
(**A**) Schematic illustration for the preparation of FA-HSA-ATO NPs. (**B**) The synergistic antitumor effect was confirmed via in vivo study. Reproduced with permission from ref. [83], Wiley, 2017.

As a candidate for drug delivery systems, albumins are desirable because of their numerous binding sites and large size [78]. Several drugs are available in the market that are encapsulated in albumin, possibly reducing the toxicity and increasing the availability of the drug (Figure 5) [79]. Among albumins, human serum albumin (HSA) and bovine serum albumin (BSA) are more suitable candidates for transporting or carrying drugs to the target site in tumor therapy [80,81]. The main biological function of HSA is to transport essential fatty acids from adipose tissues to muscles [78]. HSA as a drug carrier is more effective since it is one of the major circulating proteins in the human body, consequently avoiding immunogenic reactions [78]. An in vitro study against breast cancer cells confirmed the chemotherapeutic potential of ATO in combination with HSA in which the BSA microspheres containing ATO were prepared using chemical crosslink and solidification [68,82]. The release experiments indicated a slower release of ATO after an initial burst from the microspheres, with a cumulative release near to 95% [53]. Microspheres containing ATO and cell-penetrating peptides may have higher cellular absorption and, as a result, increased intracellular delivery [74].

### 4.3. Polymers

**Figure 6:**
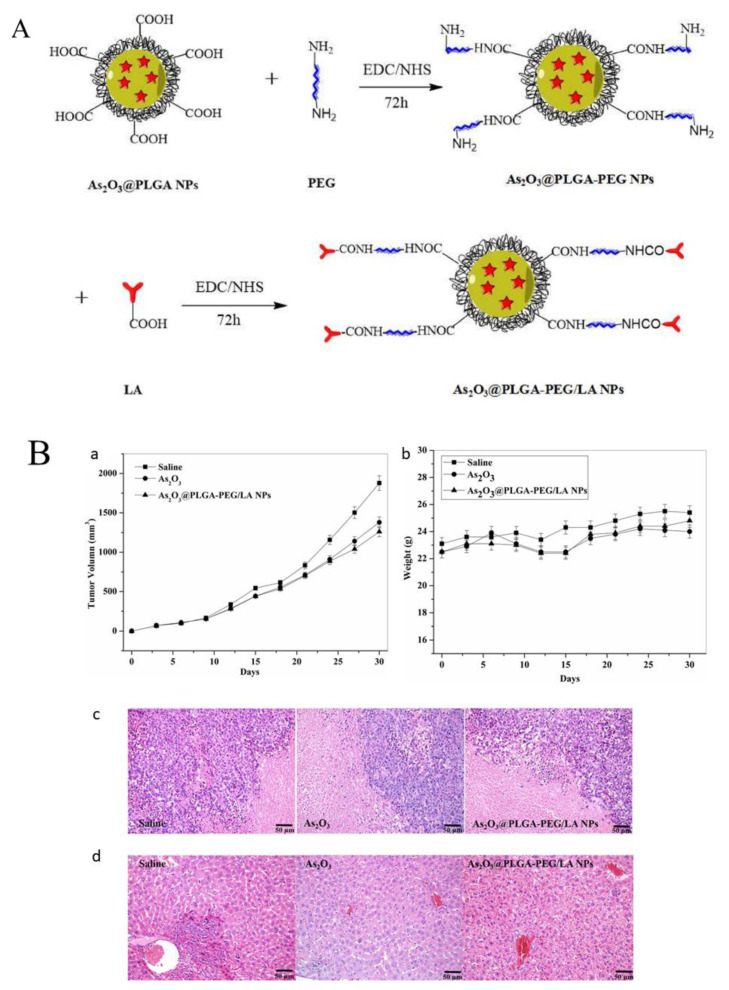
Surface modification of As_2_O_3_ @PLGA NPs. (**A**) Tumor volume curves after intravenous injection of saline, As_2_O_3_, and As_2_O_3_@PLGA-PEG/LA NPs. (**B**) Body weight curves after intravenous injection of saline, As_2_O_3_, and As_2_O_3_@PLGA-PEG/LA NPs. (**c**) Liver H&E histology images of the mice. (**d**) Tumor H&E histology images of the mice. Reproduced with permission from ref. [62], Elsevier, 2018.

Polymersomes are nanoparticles made up of block copolymers arranged in a bilayer, like liposomes, surrounded by an aqueous core with the hydrophobic polymer wall [74]. In recent years, biodegradable poly lactic-co-glycolic acid (PLGA) nano/microparticles have successfully delivered numerous chemotherapeutics [84]. Previously reported strategies indicated that PLGA-PEG NPs could be an effective nano-size delivery system of ATO for cancer treatment (Figure 6) [62]. By encapsulating ATO microcrystal with PLGA, Degang Kong et al. developed microspheres cored with extremely high dense ATO. The drug loading efficiency of ATO was 40.1%, which is 4–20 fold higher than that of reported ATO nano/microparticles [69]. These microspheres induced oxidative stress and apoptosis in hepatocellular carcinoma (HCC) cells, resulting in 80% tumor growth inhibition via locoregional delivery [69]. Song et al. found that ATO-loaded nanoparticles based on PLGA presented suitable physical stability, favorable size, moderate release rate, the highest anti-cancer effects, and cellular internalization against liver cancer [62]. In addition, Liu et al. demonstrated that arsenic nanoparticles modified with DSPE-mPEG (AsNPs@PEG) could effectively reduce the viability of different cancer cells but showed less toxicity in normal cells. Despite large doses and repeated administration, the in vivo data showed that AsNPs@PEG efficiently prevented the growth of solid tumors while remaining biocompatible in healthy tissues over the long term [70].

### 4.4. Coordination Polymer

**Figure 7:**
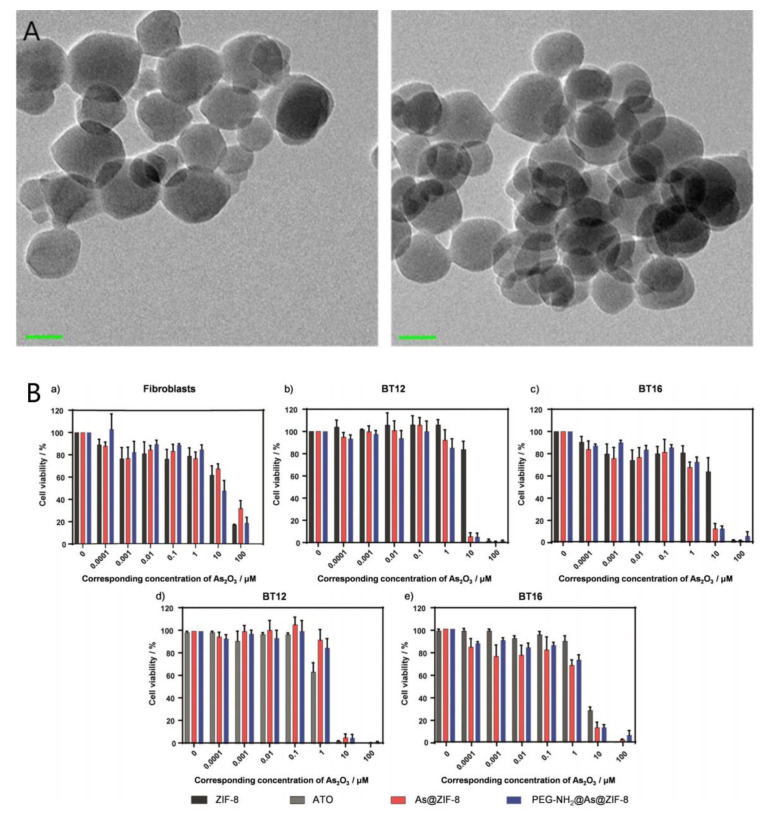
(**A**) TEM image of ZIF-8 nanoparticles before (left) and after the As drug loading, scale bar: 50 nm. (**B**) Cell viability of (**a**) fibroblasts, (**b**,**d**) BT12- and (**c**,**e**) BT16 cells after 72 h of incubation with different concentrations of ZIF-8 (black), ATO (grey), As@ZIF-8 (red) or PEG-NH_2_@As@ZIF-8 (blue). The given concentrations correspond to the As_2_O_3_ (0–100 mm) effectively loaded. Reproduced from ref. [86], Wiley-VCH, 2019.

Nanometal-organic frameworks have the properties of diverse chemical composition and structure, biodegradability, a simple and controllable preparation, high porosity, and have been widely used in drug delivery, biological imaging, gas storage, separation and catalysis, etc. (Figure 7) [85]. Romy Ettlinger et al. discovered that a Zn-based metal-organic framework called ZIF-8 (Zeolitic Imidazolate Framework) could be a promising candidate that could enable a high drug loading capacity due to its high porosity. They successfully introduced anionic As-drug to the neutral ZIF-8 via post-synthetic ligand exchange. The As@ZIF-8 nanoparticles showed a loading capacity of 74 mg of As per 1 mg of material. The As@ZIF-8 nanoparticles released only a little ATO at the neutral pH, but a complete ATO released at the more acidic pH value, making nanoparticles very desirable in cancer treatment. It could trigger specific cytotoxicity at low concentrations in rhabdoid tumor cell lines, thus drastically reducing the possible toxicity on healthy tissues. This study indicated the therapeutic potential of ATO encapsulated into ZIF-8 [71].

### 4.5. Hollow Porous Silica Nanoparticles-Based Nanovehicles

**Figure 8:**
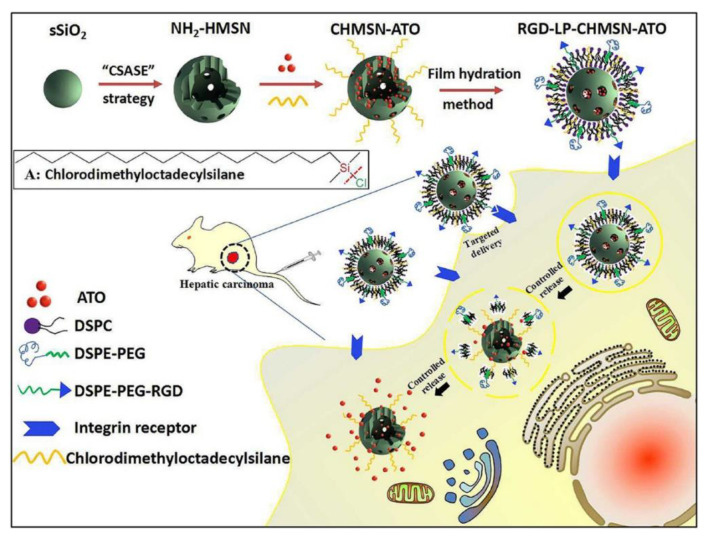
Schematic illustration for the preparation of RGD-LP-CHMSN-ATO for hepatic carcinoma therapy. Reproduced with permission from ref. [56], Elsevier, 2017.

Hollow porous silica nanoparticles (HSNs) are promising candidates for ATO delivery because of their unique and intrinsic features, such as surface modification, high stability, particle size, and excellent biocompatibility (Figure 8) [56,87]. More importantly, the hollow structure of HSNs allows the inner region to carry the inorganic drug and the outer region to be modified with targeting motifs [88]. This particular property could boost the drug loading efficacy, preserve the drug agents from environmental or enzymatic degradation and assure drug efficiency.

Chi et al. utilized HSNs as carriers to deliver ATO and achieved highly efficient treatment of solid tumors with low adverse effects. They found that HSNs loaded with ATO, prodrug manganese arsenite, significantly improved the efficacy of ATO for tumor treatment as compared to ATO alone [72]. Arsenite-loaded nanoparticle drugs can increase the cellular uptake of arsenite. This loading strategy destroyed the structure of microtubules and microfilaments and killed the circulating tumor cells efficiently [72]. Besides manganese, many other metal elements (Gd^2+^, Pt^2+^, Zn^2+^, and Fe^2+^) have also been developed to construct arsenic-metal-containing nano drugs using a metal-ion gradient loading method or reverse microemulsion approach for solid tumor therapy [89]. Wu et al. employed silica nanospheres as templates to fabricate a hollow and mesoporous ZrO_2_ nanostructure with a high specific surface area and a suitable pore size for administering ATO in a manner that allowed for precise local release. They demonstrated the safety of this method for administering high dosages of ATO. In addition, they tested this new technique in combination with microwave heat therapy. The nano-ZrO_2_ carriers used in this platform constitute a new non-invasive technique for ATO chemotherapy against HCC [65].

## 5. Combinations of ATO That Enhanced the Efficacy of Chemo Agents

With the continuous development of medical technology, the complementary strategies for disease treatment have gradually upgraded, but chemotherapy is still the main treatment for malignant tumors [90]. Anti-cancer chemotherapy drugs have undergone rapid development, and some effective anti-cancer drugs have been developed [28]. For example, molecular-targeted therapeutics have provided a superior option for systemic chemotherapy [28]. Many studies have shown that multidrug resistance (MDR) of tumor cells is the main cause of chemotherapy failure [91,92]. Simultaneously, some studies also showed that the combination of ATO with other drugs has a strong inhibitory effect on the MDR tumors (Table 2) [93,94,95,96]. Therefore, we have integrated some studies on the combination of ATO with other drugs from the last five years, hoping to enhance the knowledge of the treatment of malignant tumors in the future.

### 5.1. Combined with Molecular-Targeted Drug

Currently, molecular-targeted drugs are a popular class of tumor therapeutics compared with common drugs [109]. Molecular-targeted drugs such as tyrosine kinase inhibitors (TKIs) have more significant anti-cancer effects and lower toxicity. However, drug resistance is the main challenge for the good prognosis of cancer patients [94]. As a potential new anti-cancer drug, ATO has played a significant role in the combined treatment to reverse/slow down the occurrence of drug resistance [23,93,94,98]. Wang et al. showed that ATO combined with dasatinib induced apoptosis more pronouncedly in Philadelphia chromosome-positive acute lymphoblastic leukemia (Ph^+^ ALL) cells. Additionally, this combination repressed several genes’ expression, which is associated with a shorter survival probability in ALL patients [93,94]. Amplification/overexpression of the epidermal growth factor receptor (EGFR) gene as a signature genetic abnormality of GBM tumors can be a cause of chemo-resistance [94]. They used erlotinib as an EGFR inhibitor to increase the sensitivity of GBM cell lines to ATO treatment. The results showed that the combination of ATO with erlotinib synergistically reduced the proliferation and colony forming potential of GBM cells [94]. The combination of all-trans retinoic acid (ATRA) and ATO is effective in treating hematological malignancies. This combination therapy achieved complete remission rates of over 90% and overall survival of up to 99% [23]. Some studies suggested that ATRA combined with ATO can significantly inhibit the proliferation and promote apoptosis of NB4 cells [98]. Additionally, combining ATO with ATRA improved patients quality of life, lowered the risk of APL recurrence, and reduced its time to reach complete remission [98].

### 5.2. Combined with Chemotherapy Drugs

Many reports showed the combinatorial treatments of ATO with generic anti-cancer drugs for tumor therapy. These combinations included with cisplatin, temozolomide, silibin, paclitaxel, etoposide, vincristine, vinblastine and cytarabine [8,99,110]. Despite the rapid progress of molecular-targeted drugs, platinum-based combination chemotherapy is still an important therapy against many malignancies [28]. The adjuvant therapy of ATO with alkylating agents synergistically inhibited the proliferation of acquired drug resistance cancer cells [100]. Recently, the co-treatment of ATO with cisplatin or doxorubicin showed a synergistic inhibitory effect against leukemia cells [110]. In an in vitro study, the combination of ATO and cisplatin showed a better synergistic effect against various carcinoma cell lines than the treatment alone [96].

Arsenoplatins is another form of ATO combined with platinum drugs, small molecule complexes of an aqueous form of ATO linked chemically to Pt(II) [8]. Miodragovic et al. reported the formation of arsenoplatins, which are stable in solution, display superior anti-cancer activity, and overcome drug resistance, a major limitation of platinum drugs [99]. In addition to platinum drugs, Bureta C et al. revealed that ATO in combination with temozolomide (TMZ) or Vismodegib (VIS) significantly inhibited GBM cell growth compared to treatment alone [100]. Another combination of ATO and silibin synergistically inhibits glioma cell proliferation and induced apoptosis. This combination decreased the mRNA levels of cathepsin B, urokinase-type plasminogen activator, Bcl-2 and upregulated caspase-3 [101].

Another report showed that ATO and PTX co-delivered by nanoparticles displayed a significant synergistic effect against MCF-7 cells. This combinatorial treatment significantly affected the cell-cycle and induced apoptosis [102]. In a clinical trial on recurrent osteosarcoma and Ewing sarcoma, ATO combined with etoposide or paclitaxel achieved complete remission in five of 32 patients and partial response was observed in another six patients [23]. Furthermore, synergistic effects were shown for combining ATO with vincristine, vinblastine and lithium chloride [23]. Meister et al. identified synergistic induction of apoptosis by ATO together with several anti-microtubule agents, including vincristine and vinblastine. ATO therapy inhibited tumor growth and dramatically enhanced survival in medulloblastoma mice models. ATO also improved cytarabine chemosensitivity [23,103].

### 5.3. Combined with Other Drugs

Some chemotherapeutics that are not common in treating malignant tumors also exhibited synergistic effects when combined with ATO. Their combined application enhanced the antitumor effect of ATO. As the most important intracellular antioxidant, GSH depletion increased ATO toxicity in various cell types. This toxicity increased via mechanisms including increased oxidative stress and free active arsenic concentration, and reduction in the detoxification by GSH dependent methylation and the binding of ATO to GSH [18,26,28]. In the C6 cell line, it was discovered that butylthionine sulfoxide, an inhibitor of GSH synthesis, could decrease intracellular GSH and had a synergistic effect in combination with ATO [104]. Gartenhaus et al. reported that ascorbic acid was one such compound that depletes intracellular glutathione levels. Ascorbic acid further auto-oxidised to release H_2_O_2_, which might enhance the proapoptotic effects of ATO [111]. Other research also found that ATO and ascorbic acid (AA) effectively inhibited the viability of human colorectal cancer cells synergistically [26]. AA and ATO corporately activated caspase-3 to trigger apoptosis. This co-treatment also induced pyroptosis through the upregulation of caspase-1 [26].

A report showed that adjuvant therapy of ATO with itraconazole effectively reduced the proliferation of medulloblastoma cells through the modulating Hedgehog (Hh) pathway [23]. Non-steroidal anti-inflammatory drugs have increased cancer cells sensitivity to anti-cancer therapies such as radiotherapy, biological therapy and chemotherapy [8]. Sulindac has been reported to enhance caspase activation via ROS generation when used in combination with ATO on human lung cancer cells [105]. Similarly, indomethacin is another effective combination with ATO, which allowed a much lower concentration of ATO to carry out its anti-cancer properties [105].

Gene silencing therapy combined with ATO showed an excellent synergistic effect against malignant tumors. Zeng et al. showed a synergistic effect by silencing the mutant KRAS gene with ATO therapy on pancreatic tumor cell lines. This combination inhibited proliferation, migration, invasion and induced apoptosis in pancreatic cancer cells [107]. Blue light-emitting diode (LEDs)-based therapy has recently been shown to be a promising therapeutic approach for various malignancies [108]. Chao et al. found that combining ATO with blue LEDs had a synergistic inhibitory effect on human OS cells, which was associated with boosted ROS levels and the activation of p53 mediated by DNA damage [108].

## 6. Conclusions and Future Perspective

ATO, in combination with nano-formulations that have been reported, mostly consist of silica, polyamidoamine (PAMAM), albumin formulations, polymer micelles and liposomes-based nano-drugs. ATO is a dose-restricted anti-cancer drug that exhibits severe side effects on healthy tissues. As a result, it is crucial to monitor ATO nano-drugs’ stability and drug leakage before reaching the tumor site. Under physiological conditions, ATO produces arsenic acid (As(OH)_3_) [77]. Therefore Under physiological conditions, the ATO-loaded liposomes were frequently unstable, and large amounts of ATO would be lost within a few hours [112]. The clinical usage of liposomes to administer ATO was limited due to this characteristic. Despite the fact that ATO has been demonstrated to be a broad-spectrum anti-tumor drug, clinical outcomes have fallen short of our expectations due to high systemic toxicity and rapid renal clearance [89].

At present, the clinical efficacy of ATO could improve via the nano-drug delivery system and the combination with chemotherapeutics. In this review, we summarized the multiple anti-cancer mechanisms of ATO, novel nano-drug delivery systems, and possible combinations of ATO with chemotherapeutic agents. It is necessary to investigate the efficacy of ATO in combination with other chemo agents against various cancers further. Additionally, it is important to identify the tissue marker of solid tumors that are sensitive to ATO. Therefore, we should conduct more pre-clinical and clinical studies to reveal the anti-cancer effect of ATO that efficiently kills cancer cells with low toxicity.

## Figures and Tables

**Table 1:** Therapeutic effects of different ATO nanoparticle formulations on tumor diseases.

Nanoparticle Formulation	Experimental Models	Drug Delivery Targets	Outcome of Treatment	References
Liposome	In vitro	Via folate receptor (FR) mediated endocytosis	Significantly increased both the potency and specificity of ATO to the relatively insensitive solid tumor-derived cells	[66]
	In vitro and in vivo	Mediated by the lipoprotein receptor-related (LRP) receptor	Promoted the anti-glioma effect of ATO	[67]
Protein	In vitro	Serum albumin interacts with cell surface glycoprotein 60 receptor (albondin) and/or SPARC, leading to transcytosis	Increased cellular uptake and had better cytotoxicity	[68]
Polymers	In vitro and in vivo	Chemoembolization of tumor vessels was performed by drug elution	Inhibited tumor growth on HCC cells	[69]
	In vitro and in vivo	Via the modified with DSPE-mPEG to prolong the in vivo systemic circulation of the nanodots	Effectively reduced the viability of different cancer cells but showed less toxicity in normal cells, inhibited the growth of solid tumors	[70]
Coordination polymer	In vitro	Drug release triggered by a pH change in the vicinity of the tumour	Triggered specific cytotoxicity at low concentrations and drastically reduced the possible toxicity on healthy tissues.	[71]
Hollow porous silica nanoparticles-based nanovehicles	In vitro and in vivo	By passive targeting of the HSN through an enhanced permeability and retention effect	Significantly improves the efficacy of ATO for tumor treatment and increases the cellular uptake of arsenite.	[72]
	In vitro and in vivo	A TPP mitochondrial targeting marker was loaded to enhance mitochondrial targeting by the nanoparticles	Improves the activity of chemotherapeutics and the efficiency of hyperthermia therapy	[65]

**Table 2:** Chemopotentiating activities of ATO against various neoplastic diseases.

ATO in Combination with Chemo Agents	Experimental Models	Identified Molecular Targets and Signaling Pathways	Outcome of Combinatorial Treatment	References
Dasatinib	In vitro	Activated the UPR apoptotic IRE1/JNK/PUMA axis, neutralized the UPR ATF4-dependent anti-apoptotic axis	Increased apoptosis in both TKI-sensitive and resistant Ph^+^ ALL cell lines	[97]
Erlotinib	In vitro	Triggered ATO-induced apoptosis in GBM cell lines and increased reactive oxygen species generation	Synergistically reduced metabolic activity, proliferation and colony forming potential in treated GBM cell lines	[94]
All-trans retinoic acid (ATRA)	In vitro and In vivo	↑ Apoptosis of NB4 cells and ↓ serum IL-6 and TNF-α levels in patients with APL	Significantly inhibits the proliferation of NB4 cells and promotes their apoptosis, and reduces inflammatory responses in patients with APL	[98]
Cisplatin	In vitro	Induced apoptotic cell death	Displayed significant anti-cancer activity in a panel of human cancer cell lines and overcame the tumor-based drug resistance	[99]
Temozolomide (TMZ) and Vismodegib (VIS)	In vitro and In vivo	Damaged the DNA repair enzyme encoded in the human as O6-methylguanine-DNA methyltransferase (MGMT) and inhibited The Hedgehog (Hh) signalling pathway	Synergistically inhibited the proliferation of glioblastoma and decreased tumor growth in mice	[100]
Silibin	In vitro	Decreased the mRNA levels of cathepsin B, urokinase-type plasminogen activator, Bcl-2 and upregulated caspase-3	Synergistically inhibits glioma cell proliferation and induced apoptosis	[101]
Paclitaxel	In vitro and In vivo	Inhibited the expression of Bcl-2, caspase-7, caspase-9, cyclin B-1, and cyclin D-1; induced apoptosis	Significantly affected cell-cycle and induced apoptosis	[102]
Vincristine	In vitro	Triggered caspase-dependent apoptosis via the mitochondrial pathway	Synergistically induced apoptosis	[103]
Butylthionine sulfoxide	In vitro	Depleted intracellular GSH	Enhanced the ATO-toxicity in C6 cells	[104]
Ascorbic acid	In vitro	Activated caspase-3 to trigger apoptosis, upregulated the expression of caspase-1 and promoted formation of inflammasomes	Enhanced the proapoptotic effects of arsenic, synergistically inhibited the viability of human CRC cells	[26]
Itraconazole	In vivo	Modulating Hedgehog (Hh) pathway	Effectively reduced tumor growth of medulloblastoma cells	[23]
Sulindac	In vitro	Increased the catalytic activity of caspase-3, -8, and -9 along with induction of Fas/FasL expression and cytosolic release of cytochrome c	Synergistically enhanced cytotoxicity to NCI-H157 lung cancer cells	[105]
Indomethacin	In vitro	Activation of ERK and p38 pathways, considerably high Caspase-3 activity	Exerted a very potent in vitro cytotoxic effect against A549 lung cancer cells	[106]
SiRNA-directed Kras oncogene silencing	In vitro and In vivo	Down-regulation of the mutant Kras gene by siRNA and tumor growth inhibition of arsenic	Inhibited proliferative, migratory and invasive pancreatic cancer cells, and substantially improved the apoptotic effect	[107]
Blue LED irradiation	In vitro	Increased ROS accumulation, DNA damaged mediated p53 activation	Significantly decreased the percentages of proliferative cells, and increased apoptotic rate on human osteosarcoma	[108]

## Data Availability

Not applicable.

## Abbreviations

A2-PEG-LP@CaAs, angiopep-2-modified calcium arsenite-loaded liposomes; AA, Ascorbic acid; APL, acute promyelocytic leukemia; As, Arsenic; ATO, Arsenic trioxide; ATRA, all-trans retinoic acid; BSA, bovine serum albumin; CSCs, cancer stem cells; Cu (OAc)_2_, copper acetate; Dll4/Notch-1, delta-like canonical Notch ligand 4; ECM, extracellular matrix; EGFR, epidermal growth factor receptor; EPR, enhanced permeability and retention; FasL, Fas ligand; FDA, Food and Drug Administration; FGF-2, fibroblast growth factor-2; GBM, Glioblastoma multiform; GSH, glutathione; HCC, hepatocellular carcinoma; Hh, Hedgehog; HSA, human serum albumin; HSNs, hollow porous silica nanoparticles; HUVECs, human umbilical vein endothelial cells; IARC, International Agency for Research on Cancer; iAs, inorganic arsenic compounds; LEDs, light emitting diodes; LRP, lipoprotein receptor-related; MDR, multidrug resistance; MMD, mitochondrial membrane depolarization; MMPs, matrix metalloproteinase; MOMP, mitochondrial outer membrane permeabilization; PDGF, platelet-derived growth factor; PLGA, poly lactic-co-glycolic acid; ROS, reactive oxygen species; RTS, realgar transforming solution; SCLC, small cell lung cancer; TCM, traditional Chinese medicine; TKIs, tyrosine kinase inhibitors; TMZ, temozolomide; TNF, tumor necrosis factor; TRAIL, tumor necrosis factor-related apoptosis-inducing ligand; VEGF, vascular endothelial growth factor; VIS, Vismodegib; XIAP, X-linked inhibitor of apoptosis protein; ZIF-8, Zeolitic Imidazolate Framework.
